# Hidden Cardiac Channelopathies in Children Presenting with Syncope and Seizure-like Events

**DOI:** 10.3390/children13050669

**Published:** 2026-05-12

**Authors:** Funda Aytekin Güvenir, Senem Özgür

**Affiliations:** 1 Department of Pediatric Allergy and Immunology, Ankara Bilkent City Hospital, Ankara 06800, Turkey; 2 Department of Pediatric Cardiology, Ankara Etlik City Hospital, Ankara 06170, Turkey; drsenemozgur@hotmail.com

**Keywords:** syncope, seizures, cardiac channelopathies, long QT syndrome, pediatric

## Abstract

**Highlights:**

**What are the main findings?**

- Cardiac channelopathies were identified in 4.9% of children presenting with syncope or seizure-like events.
- Clinical triggers, ventricular arrhythmias, and abnormal exercise test findings were significantly more frequent in patients with channelopathies.

**What are the implications of the main finding?**

- Cardiac channelopathies should be considered in the differential diagnosis of pediatric syncope and seizure-like presentations to avoid misdiagnosis.
- Early recognition through detailed history and electrocardiographic evaluation may reduce the risk of life-threatening arrhythmias and sudden cardiac death.

**Abstract:**

**Background:** Cardiac channelopathies are rare but potentially life-threatening disorders that may present with syncope or seizure-like episodes in children, often leading to misdiagnosis and delayed recognition. Other arrhythmia-associated cardiac conditions may also present with similar clinical manifestations and require careful cardiac evaluation. **Objective:** To evaluate the prevalence of cardiac channelopathies and inherited arrhythmogenic cardiac disorders in pediatric patients presenting with syncope and seizure-like events and to identify associated clinical and electrocardiographic features. **Methods:** This retrospective cross-sectional study included pediatric patients presenting with syncope, presyncope, seizures, or seizure-like episodes who underwent cardiac evaluation at Ankara Dr. Sami Ulus Maternity and Children’s Health and Diseases Training and Research Hospital between January 2015 and April 2019. Cardiac evaluation was performed using a standard 12-lead electrocardiogram and was complemented by additional investigations, including 24 h Holter monitoring, exercise testing, pharmacological provocation, electrophysiological studies, and genetic analysis, when clinically indicated. Demographic, clinical, and diagnostic parameters were systematically evaluated. **Results:** A total of 363 patients were included in the final analysis. The mean age was 12.2 ± 4.7 years, and 58.7% were female. The most common diagnosis was vasovagal syncope (*n* = 160, 44.1%), followed by epilepsy (*n* = 53, 14.6%). Cardiac channelopathies, and arrhythmogenic right ventricular dysplasia (ARVD) were identified in 18 patients, corresponding to 4.9% of the pediatric cardiology-evaluated patients and 0.82% of the initial screened population. These diagnoses included long QT syndrome (*n* = 8), Brugada syndrome (*n* = 3), short QT syndrome (*n* = 3), catecholaminergic polymorphic ventricular tachycardia (*n* = 2), ARVD (*n* = 1), and malignant-type early repolarization (*n* = 1). Compared with other patients, those with cardiac channelopathies, malignant-type early repolarization, and ARVD more frequently had exercise-related triggers (*p* < 0.001), ventricular extrasystoles and ventricular tachycardia (*p* < 0.001), and abnormal exercise test findings (*p* < 0.001). **Conclusions:** Cardiac channelopathies are not uncommon in pediatric patients presenting with syncope and seizure-like events and should be considered in the differential diagnosis. Clinical triggers, family history, and electrocardiographic abnormalities may serve as important clues for early identification. A multidisciplinary approach, including detailed cardiac evaluation, is essential to prevent misdiagnosis and reduce the risk of sudden cardiac death.

## 1. Introduction

Cardiac channelopathies are primary ion channel disorders that occur in structurally normal hearts and can result in sudden cardiac death. They are rare but important causes of syncope and seizure-like episodes in childhood [1,2].

These diseases are mostly associated with mutations in genes encoding cardiac ion channels or related regulatory proteins. Gain-of-function or loss-of-function mutations in calcium, sodium, and potassium channels disrupt the duration and physiology of the action potential, predisposing patients to ventricular arrhythmias [3,4,5].

Hereditary arrhythmia syndromes such as long QT syndrome (LQTS), Brugada syndrome (BrS), catecholaminergic polymorphic ventricular tachycardia (CPVT), and arrhythmogenic right ventricular dysplasia (ARVD) can cause cerebral hypoperfusion through ventricular arrhythmias and present with convulsion-like motor activity [6]. This condition is easily confused with epilepsy, especially in the pediatric age group, and can lead to delayed diagnosis [4].

Mutations in ion channel genes that affect both neuronal and cardiac excitability have been shown to cause both seizure and arrhythmia phenotypes. This bidirectional relationship highlights the critical importance of cardiac evaluation in unexplained seizure-like episodes [7,8].

This study aimed to determine the prevalence of cardiac channelopathies in patients presenting during childhood with syncope, presyncope, seizures, or seizure-like activity and to evaluate these patients in terms of clinical findings, triggering factors, and warning signs.

## 2. Materials and Method

### 2.1. Study Design

This retrospective, cross-sectional, descriptive study was conducted in the Pediatric Cardiology, Pediatric Neurology, and Pediatric Emergency Departments of Ankara Dr. Sami Ulus Maternity and Children’s Health and Diseases Training and Research Hospital. The study included pediatric patients who presented between 1 January 2015, and 30 April 2019, with syncope, presyncope, seizures, or seizure-like events and were evaluated by the Department of Pediatric Cardiology. Hospital records were screened retrospectively using ICD-10 diagnostic codes, including G40, R55, and I44–I49, followed by a detailed review of medical records to confirm eligibility. From this source population, consecutive patients who underwent cardiac evaluation by the pediatric cardiology department using appropriate diagnostic tests and who had sufficient clinical data were included. Patients with incomplete medical records or insufficient information to establish the final diagnosis were excluded.

Demographic data, presenting complaints, clinical findings, ECG results, and, when available, 24 h Holter monitoring results, cardiac provocation test findings, electrophysiological study (EPS) findings, and genetic results were recorded. All patients included in the study underwent a standard 12-lead ECG. Twenty-four-hour Holter monitoring, cardiac provocation tests, and EPS were performed in selected cases based on clinical necessity.

### 2.2. Electrocardiogram

Resting 12-lead ECGs were performed in all cases according to the electrocardiography standardization and recording recommendations of the American Heart Association Electrocardiography and Arrhythmias Committee, Council on Clinical Cardiology, American College of Cardiology Foundation, and Heart Rhythm Society [9]. Heart rate, rhythm, PR interval, QRS duration, axis, and repolarization characteristics were evaluated on ECG recordings. The QT interval was measured to the end of the T wave, and the heart rate-corrected QT (QTc) value was calculated using the Bazett formula (QTc = QT/√RR) [9].

### 2.3. Holter Monitoring

In this study, patients were connected to a portable Holter recording device via biopotential electrodes placed on the chest wall, and continuous ambulatory electrocardiographic recordings were obtained for 24 h during daily activities and sleep. Holter recordings were digitally analyzed and evaluated for mean, minimum, and maximum heart rate; presence of sinus rhythm; supraventricular and ventricular premature beats; episodes of tachycardia and bradycardia; pauses; and conduction disturbances [10]. Additionally, the recordings were reviewed in detail by an experienced physician for clinically significant rhythm abnormalities and symptom–rhythm correlation.

### 2.4. Exercise Provocation Test

Tests were performed on a treadmill according to standard protocols. During exercise and the recovery phase, patients were continuously monitored with 12-lead electrocardiography and blood pressure monitoring. During the test, heart rate response, achievement of age-predicted maximum heart rate, chronotropic response, exercise-induced supraventricular or ventricular arrhythmias, conduction disturbances, and electrocardiographic changes consistent with ischemia were evaluated. The test was terminated if clinically or electrocardiographically significant findings were detected. Pathological exercise test findings were defined as clinically significant abnormalities observed during exercise or recovery, including frequent or complex exercise-induced ventricular extrasystoles, ventricular tachycardia, abnormal QTc adaptation or dynamic QTc changes, and significant repolarization abnormalities [11].

### 2.5. Pharmacological Provocation Test

A pharmacological provocation test with ajmaline was performed in cases suspected of Brugada syndrome. Tests were conducted under continuous electrocardiographic and hemodynamic monitoring, with resuscitation equipment readily available. ECGs were recorded serially during ajmaline infusion. Positivity of the ajmaline provocation test was evaluated according to international consensus criteria. Accordingly, the development of a type 1 Brugada pattern characterized by ≥2 mm coved ST-segment elevation in the right precordial leads (V1–V3), particularly in leads V1 and V2, during the test was considered a positive response. Additionally, the occurrence of clinically significant ventricular arrhythmia during the test was also considered a positive result [12].

### 2.6. Electrophysiological Study

EPS was performed in selected cases in which advanced evaluation was considered necessary because of high-risk arrhythmia or the presence of an accessory pathway in the etiology of syncope/presyncope. EPS was conducted using standard intracardiac catheters under fluoroscopy. During the study, atrioventricular conduction properties, the presence and conduction characteristics of accessory pathways, arrhythmia inducibility, and the effective refractory periods of the accessory pathway, atrial tissue, and ventricular tissue were evaluated [13].

### 2.7. Cardiac Channelopathy and ARVD Diagnoses

#### 2.7.1. Long QT Syndrome

The diagnosis of LQTS was based on the combined evaluation of electrocardiographic findings, clinical symptoms, and family history. The QT interval was measured on resting 12-lead electrocardiography based on the end of the T wave, and the QTc value was calculated using the Bazett formula. The Schwartz scoring system, which includes QTc duration, T-wave morphology, syncope history, and family history of sudden cardiac death, was used to determine diagnostic probability; cases with a Schwartz score ≥ 3.5 were considered highly probable for LQTS. In selected cases in which clinical suspicion persisted and resting QTc values were normal or borderline, diagnostic evaluation was supported by exercise provocation testing and genetic studies [14].

#### 2.7.2. Short QT Syndrome (SQTS)

The diagnosis of SQTS was assessed using the diagnostic scoring system proposed by Gollob et al. The score incorporates electrocardiographic findings, clinical history, family history, and genetic results. QTc was calculated using Bazett’s formula, and points were assigned as follows: QTc ≤ 370 ms (1 point), ≤350 ms (2 points), and ≤330 ms (3 points). A J-point–Tpeak interval ≤ 120 ms in the precordial lead with the highest T-wave amplitude was assigned 1 point. Additional points were allocated for a history of cardiac arrest, documented polymorphic ventricular tachycardia/ventricular fibrillation, unexplained syncope, atrial fibrillation, a positive family history, or the presence of a pathogenic mutation in previously established SQTS-associated genes. A total score of ≥4 was considered high probability, 3 intermediate probability, and ≤2 low probability for SQTS. QT measurements were evaluated in the absence of reversible causes of QT shortening [15].

#### 2.7.3. Brugada Syndrome

The diagnosis of Brugada syndrome was based on the detection of a type 1 Brugada pattern, characterized by ≥2 mm coved ST-segment elevation in the right precordial leads (V1–V2) on resting 12-lead electrocardiography. Electrocardiographic evaluation was supported by recording right precordial leads from higher intercostal spaces when necessary and by repeated ECG recordings. In selected cases in which a spontaneous type 1 pattern was not observed but clinical suspicion was high, diagnostic evaluation was performed using a pharmacological provocation test with a sodium channel blocker (ajmaline/procainamide). Type 2 and type 3 Brugada patterns were not considered diagnostic on their own; further evaluation was planned in these cases. Before diagnosis, secondary causes that could lead to Brugada phenocopy (ischemia, metabolic disorders, structural heart disease) were excluded [16,17].

#### 2.7.4. Catecholaminergic Polymorphic Ventricular Tachycardia

The diagnostic evaluation of CPVT was based on the presence of ventricular arrhythmia triggered by exercise or emotional stress in patients without structural heart disease and with normal resting electrocardiograms. In cases with clinical suspicion, an exercise provocation test was performed, and bidirectional or polymorphic ventricular tachycardia, or frequent ventricular premature beats occurring with increased heart rate, were considered diagnostically significant. In selected cases in which exercise testing could not be performed or was not diagnostic, the presence of arrhythmia was assessed by Holter monitoring. The diagnosis of CPVT was made based on clinical findings, exercise-induced arrhythmic response, and, in appropriate cases, the presence of pathogenic genetic variants (particularly RYR2, CASQ2, and related genes) [18].

#### 2.7.5. Arrhythmogenic Right Ventricular Dysplasia

The diagnosis of ARVD was made based on the American Heart Association Revised Task Force Criteria. Diagnostic evaluation included right ventricular structural and functional abnormalities (echocardiography and/or cardiac magnetic resonance imaging), tissue characterization findings (particularly evidence of fibrosis or scarring on cardiac magnetic resonance imaging), electrocardiographic findings of repolarization and depolarization abnormalities, documentation of ventricular arrhythmia (presence of ventricular premature beats and/or ventricular tachycardia by Holter monitoring and/or electrocardiography), and family history and/or the presence of pathogenic genetic variants [19].

Ethical committee approval was obtained (No: 2012-KAHK-151955). All procedures were conducted in accordance with the principles of the Declaration of Helsinki. Informed consent was obtained from the patients’ relatives before diagnostic and provocation tests were performed.

### 2.8. Statistical Analyses

Data were analyzed using SPSS version 25.0 (SPSS Inc., Chicago, IL, USA). Categorical variables were expressed as numbers and percentages, whereas continuous variables were expressed as mean ± standard deviation. The normality of continuous data was assessed based on skewness and kurtosis coefficients. For pairwise comparisons, the independent-samples *t*-test was used for normally distributed numerical data, whereas the Mann–Whitney U test was used for non-normally distributed data. The chi-square test was used to compare categorical variables. When the assumptions for the chi-square test were not met, Fisher’s exact test was used. Statistical significance was set at *p* < 0.05.

## 3. Results

### 3.1. Demographic and Clinical Characteristics

**Figure 1:**
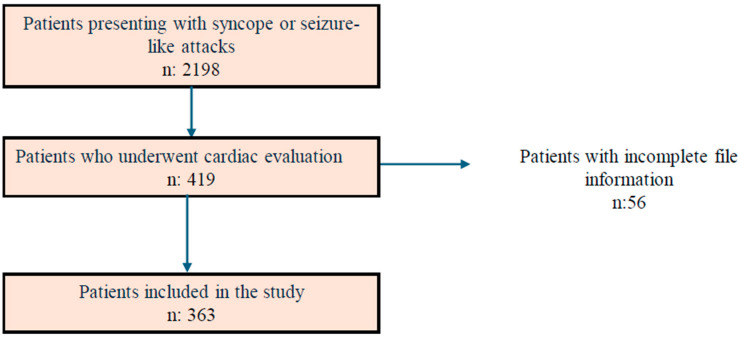
Flowchart of Patient Selection Among Children Presenting with Syncope and Seizure-Like Events.

During the study period, a total of 2198 patients presenting with syncope, seizures, and seizure-like attacks were evaluated. Of these, 419 underwent cardiologic evaluation, including ECG, echocardiography, and 24 h Holter monitoring. Fifty-six patients were excluded because of missing information. The final analysis included 363 patients (Figure 1). The mean age of the patients was 12.2 ± 4.7 years, and 213 were female (58.7%).

The most common diagnosis was vasovagal syncope, observed in 160 cases (44.1%, 160/363), followed by epilepsy in 53 patients (14.6%, 53/363). Cardiac channelopathy, malignant-type early repolarization and ARVD were detected in 18 patients, corresponding to 4.9% (18/363) of the pediatric cardiology-evaluated patients and 0.82% (18/2198) of the initial screened population.

The most common diagnosis was vasovagal syncope, observed in 160 cases (44.1%). Epilepsy was diagnosed in 53 patients (14.6%). Cardiac channelopathy and malignant-type early repolarization were detected in 18 cases (4.9%).

Echocardiographic evaluation revealed abnormal findings in 17.3% of patients, with valvular pathologies being the most common (*n* = 45, 12.3%). Regarding triggers, situational and orthostatic triggers were the most commonly observed, present in 155 patients (42.7%) (Table 1).

### 3.2. Patients with Cardiac Channelopathy, Malignant-Type Early Repolarization and ARVD

Among the 18 patients diagnosed with cardiac channelopathy and ARVD, the mean age was 12.7 ± 4.4 years, and 11 (61.1%) were male. The most common channelopathy was LQTS (*n* = 8), followed by Brugada syndrome (*n* = 3), short QT syndrome (*n* = 3), CPVT (*n* = 2), ARVD (*n* = 1), and malignant-type early repolarization (*n* = 1) (Table 2). Fifteen patients (83.3%) had an identifiable triggering factor for their symptoms; the most common was exercise (*n* = 11). Abnormal ECG findings were detected in family members of five patients (Table 2).

### 3.3. Long QT Syndrome

Eight patients were diagnosed with LQTS. Five patients were female, and the mean age was 11 ± 6.3 years. Three had a family history of sudden cardiac death, and all patients described symptoms triggered by exertion. One patient presented with complete AV block. Echocardiographic evaluation revealed normal findings in six patients, mitral regurgitation in one patient, and mitral regurgitation with mitral valve prolapse in another patient. Arrhythmogenic bileaflet mitral valve prolapse was considered in the patient with mitral valve prolapse. An electroencephalogram (EEG) in one patient revealed a focal epileptic focus in the left frontotemporal region. Three patients had sensorineural deafness and were evaluated as having Jervell–Lange Nielsen syndrome. Genetic analysis revealed heterozygous mutations in the *CACNA1C* gene in two patients and in the *CACNB2* gene in one patient.

### 3.4. Short QT Syndrome

Three patients were diagnosed with SQTS. One patient was a 16-year-old male who presented with symptoms at rest. His ECG showed a short QT interval along with features of early repolarization. The remaining two patients were 16- and 14-year-old males, both with a history of exertional syncope.

### 3.5. Catecholaminergic Polymorphic Ventricular Tachycardia

Two patients were diagnosed with CPVT—a 12-year-old female and a 4-year-old male. In both patients, symptoms were triggered by exertion. The 12-year-old patient had a family history of sudden cardiac death and had previously been followed with a diagnosis of epilepsy. A *CASQ2* mutation was detected in this patient. Echocardiographic evaluation was normal in both patients.

### 3.6. Brugada Syndrome

Three male patients aged 14, 15, and 17 years were diagnosed with Brugada syndrome. Symptoms occurred with fever in one patient and at rest in two patients. Mitral regurgitation was detected on echocardiography in two patients. Pharmacological provocation testing with ajmaline was performed in all three patients, and a Brugada ECG pattern was observed in all of them, contributing to diagnostic confirmation. An *SCN5A* mutation was detected in one patient. A variant of uncertain significance was detected in the *KCNH2* gene in one patient. A type 2 Brugada pattern was identified on the ECG of this patient’s father.

### 3.7. Arrhythmogenic Right Ventricular Dysplasia

One patient, a 16-year-old female, was diagnosed with ARVD. She had a history of intensive care unit admission because of ventricular tachycardia attacks and had an implantable cardioverter defibrillator.

### 3.8. Early Repolarization and Other Rhythm Disorders

Malignant-type early repolarization was identified in one patient, a 16-year-old male with a history of three episodes of seizure-like activity. His family history was notable for sudden cardiac death in his sister. Wolff–Parkinson–White syndrome was detected in five patients (1.4%).

### 3.9. Comparison of the Channelopathy and ARVD Group with Other Patients

When patients in the channelopathy and ARVD group were compared with other patients, no significant differences were found in age or sex distribution. However, the channelopathy group more frequently had exercise-related triggers, echocardiographic pathology (predominantly trivial), abnormal family ECG findings, abnormal exercise test results, ventricular extrasystoles, ventricular tachycardia (*p* < 0.01), and pathological T-wave morphology (*p* = 0.03). There was no significant difference between the groups in mean heart rate (Table 3).

Among the 59 patients with exercise-related triggers, 11 were diagnosed with cardiac channelopathy and ARVD, whereas 48 had other diagnoses. The positive and negative predictive values of exercise-related triggers were 18.6% and 97.7%, respectively.

## 4. Discussion

Cardiac channelopathies are rare in the general population but may lead to fatal outcomes. They may present with syncope or seizure-like episodes due to transient cerebral hypoperfusion and therefore represent an important consideration in the differential diagnosis of such clinical presentations. In the present study, we investigated the prevalence of cardiac channelopathies in pediatric patients presenting with syncope, seizure, or syncope-like complaints. Cardiac channelopathies, malignant-type early repolarization and ARVD were identified in 0.82% of the initial screened population presenting with syncope, seizure, or seizure-like events, and in 4.9% of the pediatric cardiology-evaluated patients. The higher frequency observed among patients who underwent cardiac evaluation may reflect the selected nature of this subgroup, as well as the tertiary referral characteristics of our center.

In patients with channelopathies, exercise-induced symptoms, were observed significantly more frequently. Specifically, exercise, emotional stress, and fever play a decisive role in the onset of symptoms and arrhythmias in different subtypes of channelopathies. While exercise-induced symptoms are markedly more common in LQT1 and CPVT, symptoms in Brugada syndrome are more commonly triggered by fever than by exercise [14,16,18]. In our cohort, exercise-related symptoms showed a high negative predictive value for cardiac channelopathy and ARVD, suggesting that the absence of exercise-related triggers may be associated with a lower probability of channelopathy. These differences in triggers should be considered in the diagnostic approach, risk stratification, and planning of exercise restrictions for patients.

All cardiac channelopathies can be associated with malignant arrhythmias such as polymorphic ventricular tachycardia, torsade de pointes, and ventricular fibrillation, and carry a high risk of syncope and sudden cardiac death. These syndromes are responsible for a significant proportion of sudden cardiac deaths, particularly in young individuals without structural heart disease [20,21]. Similarly, in our study, the significantly higher incidence of ventricular arrhythmias in patients diagnosed with channelopathy compared with other patient groups supports the high arrhythmic risk in this population.

Although it is often stated that cardiac channelopathies are not associated with structural heart disease [21], valvular pathologies were detected significantly more frequently in patients diagnosed with channelopathy in our study. This finding may partly reflect disturbances in heart rate regulation related to autonomic nervous system dysfunction in the channelopathy group. In addition, it may also be explained by the overlap between electrical and structural abnormalities observed in certain arrhythmogenic conditions. For example, disorders such as arrhythmogenic cardiomyopathy or arrhythmogenic mitral valve prolapse may involve both electrophysiological abnormalities and structural changes that contribute to arrhythmogenesis [22,23]. In light of these findings, echocardiographic evaluation appears important in patients with suspected channelopathy to identify accompanying structural abnormalities and allow a more comprehensive risk assessment.

In our study, the significantly higher rate of electrocardiographic abnormalities in family members of patients diagnosed with channelopathy compared with other patient groups is consistent with the hereditary nature of these diseases. This result highlights the critical importance of family-based assessments in predicting sudden cardiac death risk [24], in addition to aiding diagnosis. Detailed questioning of family history and electrocardiographic screening of first-degree relatives are indispensable components of clinical evaluation in cardiac channelopathies.

LQTS was the most frequently diagnosed cardiac channelopathy, which is consistent with the literature [25]. The fact that symptoms were triggered by exercise in all cases diagnosed with LQTS indicates that the QT interval should be carefully evaluated in cases of syncope or seizure-like activity developing after exertion, although in some cases, ventricular arrhythmias are triggered at rest. However, the QT interval may be normal or borderline on resting electrocardiography, and dynamic QT changes may occur [26]. The observation of QT interval prolongation and shortening in one case in our study supports that a single resting ECG is not sufficient to rule out LQTS.

The presence of sensorineural deafness in some cases diagnosed with LQTS is suggestive of Jervell–Lange Nielsen syndrome and indicates that cardiac channelopathies may exhibit multisystem features [27]. Furthermore, the detection of epileptiform EEG findings in one case suggests a phenotypic overlap due to the common expression of cardiac and neuronal ion channels. This finding indicates that many channelopathies, including long QT syndrome, should be reconsidered in patients diagnosed with epilepsy who present with atypical clinical features or are unresponsive to treatment.

Brugada syndrome is a rare channelopathy with a marked male predominance; this is thought to be related to the role of hormones, particularly testosterone, in phenotypic expression [28]. The fact that all patients diagnosed with Brugada syndrome in our study were postpubertal males is consistent with the literature. The triggering of symptoms by fever and rest reflects the typical clinical features of Brugada syndrome. These findings also highlight the importance of considering Brugada syndrome in patients presenting with syncope or seizure-like events during febrile episodes. Because fever is known to increase the likelihood of arrhythmic events in Brugada syndrome, particular attention should be paid to the family history of arrhythmia or sudden cardiac death in patients presenting with febrile convulsions. In our study, pharmacological provocation testing with ajmaline was performed in all three patients diagnosed with Brugada syndrome, and a Brugada ECG pattern was observed in all three, contributing to diagnostic confirmation. Therefore, upper right precordial lead ECG recordings and, when necessary, pharmacological provocation tests may play an important role in diagnosis, even when the initial ECG appears normal [12].

Although CPVT is a rare channelopathy, it is considered high risk for sudden cardiac death because it is detected with similar frequency to LQTS in autopsy series [29]. In our study, the prevalence of CPVT was 0.5%, which is consistent with the literature and indicates that it is less common than LQTS and Brugada syndrome. The fact that one of the two patients diagnosed presented with cardiac arrest highlights the severe and life-threatening clinical presentation of CPVT. In both cases, symptoms were triggered by exercise. In CPVT, where the resting ECG may be normal, the detection of polymorphic or bidirectional ventricular tachycardia during exercise testing is diagnostic [30]. Therefore, in patients presenting with exercise-induced syncope or seizure-like activity, an exercise test should always be included in the diagnostic process, even if the resting ECG is normal.

Early repolarization pattern is an electrocardiographic finding that can be seen in the general population; however, some subtypes may be associated with malignant ventricular arrhythmias [31]. The fact that malignant features were present in some of our cases with early repolarization and that these patients presented with atrial fibrillation or ventricular tachycardia indicates that this pattern should not always be considered benign. Furthermore, the observation of early repolarization findings in a case diagnosed with SQTS supports the possible relationship between these two conditions reported in the literature [32].

Apart from cardiac channelopathies and ARVD, cases diagnosed with WPW syndrome, one of the preexcitation syndromes, were also identified. Although rare, WPW is a condition that can present with syncope and sudden cardiac death, and it was observed in our study at a rate higher than that reported in the literature [33]. This indicates that electrocardiograms of patients presenting with syncope and seizure-like activity should also be carefully evaluated for preexcitation findings, particularly delta waves.

The majority of patients in our study had a diagnosis of vasovagal syncope, which is consistent with the literature. The presence of typical triggers such as prolonged fasting, standing, sudden postural changes, and emotional stimuli, accompanied by prodromal symptoms, supports the diagnosis in this patient group [34]. However, it should be noted that some prodromal complaints, such as palpitations and anxiety, can also be seen in cardiac channelopathies. Furthermore, patients with Brugada syndrome have an increased tendency toward vasovagal syncope compared with the general population. Therefore, even in patients with a suspected diagnosis of vasovagal syncope, it is important to perform an electrocardiographic evaluation to rule out cardiac syncope and to plan further cardiac investigation when necessary.

This study has some limitations. First, due to its retrospective design, data were obtained from patient records, and some cases were excluded because of incomplete clinical information. Furthermore, the fact that genetic analysis results for all patients had not yet been completed limited detailed evaluation of genotype–phenotype relationships in channelopathies. Another limitation is the potential for selection bias related to the retrospective design of the study. The study included only patients who were referred to and evaluated by the pediatric cardiology department. Patients in whom an apparent non-cardiac etiology was identified by the first evaluating clinician, or those who were not considered to require cardiological assessment, were not routinely referred for cardiac evaluation. Therefore, our findings should be interpreted as reflecting a clinically selected subgroup rather than the entire population of children presenting with syncope or seizure-like events. Diagnostic testing was not uniform across all patients, and advanced investigations such as exercise testing, pharmacological provocation testing, electrophysiological study, and genetic analysis were performed according to clinical indication. Because syncope, seizure-like events, and epilepsy may overlap clinically, potential misclassification between neurological and cardiac diagnoses cannot be completely excluded in a retrospective study. The number of patients diagnosed with cardiac channelopathies, malignant-type early repolarization, and ARVD was relatively small, which limits statistical power and the reliability of comparative analyses. In addition, QTc was calculated using Bazett’s formula; although widely used in clinical practice, this method may be affected by heart rate variability and may have limitations in pediatric populations.

The most important strength of our study is the evaluation of a large pediatric patient population presenting with syncope, seizures, and seizure-like activity. The combined analysis of electrocardiography, Holter monitoring, exercise and pharmacological provocation tests, and echocardiographic findings ensured a comprehensive assessment of cardiac causes. Furthermore, the comparative analysis of the channelopathy group with other patient groups contributed to the identification of clinical and electrocardiographic clues useful in the differential diagnosis.

## 5. Conclusions

This study demonstrates that cardiac channelopathies can occur with non-negligible frequency in pediatric patients presenting with seizures, syncope, and seizure-like activity, and can potentially lead to life-threatening arrhythmias. Therefore, in patients evaluated with a preliminary diagnosis of seizure or syncope, a purely neurological approach should not suffice; a detailed history, family history, and electrocardiographic evaluation should be integral parts of the diagnostic process.

## Figures and Tables

**Table 1:** Demographic Features, Clinical Presentations, and Diagnoses of the Study Population.

Parameters, *n* = 363	n (%)
Age at admission, mean ± SD, years	12.27 ± 4.24
Gender	
Female	213 (58.7)
Diagnoses	
Vasovagal syncope	160 (44.1)
Epilepsy	53 (14.6)
Migraine	29 (7.9)
Nonepileptic paroxysmal events	21 (5.8)
AV block	21 (5.8)
Conversion	19 (5.2)
Cardiac channelopathy, malign type early repolarization, ARVD	18 (4.9)
VES, SVES	16 (4.4)
Borderline LQT	8 (2.2)
WPW	5 (1.4)
Sinus node dysfunction	5 (1.4)
Hypoglycemia	3 (0.8)
Benign type early repolarization	3 (0.8)
Sinus tachycardia	2 (0.6)
Echocardiographic abnormalities	56 (15.4)
Valve pathologies	45 (12.3)
Mitral regurgitation	22 (6.1)
Mitral valve prolapse	16 (4.4)
Bicuspid aortic valve	3 (0.8)
Aortic regurgitation	2 (0.5)
Tricuspid regurgitation	2 (0.05)
Ventricular hypertrophy	6 (1.6)
Prominent ventricular trabeculation	5 (1.3)
Triggers	256 (70.5)
Situational and orthostatic triggers	155 (42.7)
Exercise	59 (16.3)
Emotional stress	28 (7.7)
Sleep	7 (1.9)
Fever	7 (1.9)

AV block, atrioventricular block; ARVD, arrhythmogenic right ventricular dysplasia; LQT, long QT; SVES, supraventricular extrasystole; VES, ventricular extrasystole; WPW, Wolff–Parkinson–White syndrome.

**Table 2:** Characteristics of Patients with Cardiac Channelopathy and ARVD.

Parameters, *n* = 18	n (%)
Age at presentation, mean ± SD, years	12.7 ± 4.4
Channelopathy diagnoses	
Long QT syndrome	8 (44.4)
Short QT Syndrome	3 (16.6)
Brugada syndrome	3 (16.6)
CPVT	2 (11.1)
Malign type early repolarisation	1 (5.5)
Arrhythmogenic Right Ventricular Dysplasia	1 (5.5)
Gender	
Male	11 (52.3)
Triggering Factor	15 (83.3)
Exercise	11
Fever	2
Situational and orthostatic triggers	1
Sleep	1
ECHO results	
Normal	11 (52.3)
Mitral regurgitation	4 (22.2)
Tricuspid regurgitation	1 (5.5)
Right ventricular cardiomyopathy	1 (5.5)
Patent ductus arteriosus	1 (5.5)
Abnormal family ECG findings	5 (27.7)

ECG, electrocardiogram; CPVT, catecholaminergic polymorphic ventricular tachycardia.

**Table 3:** Comparison of Clinical and Electrocardiographic Features Between Patients with Cardiac Channelopathy/ARVD and Other Patients.

Variable	Channelopathy/ARVD (*n* = 18)	Others (*n* = 345)	*p*
Age, years, mean, SD	12.7 ± 4.4	12.4 ±4.5	0.35
Gender			
Female, n (%)	7 (38.8)	206 (60.1)	0.29
Exercise-related trigger, n (%)	11 (61.1)	48 (13.9)	<0.001
Echocardiographic abnormalities	10 (55.5)	46 (13.3)	<0.001
Pathology in family ECG, n (%)	5 (27.7)	6 (1.7)	0.001
Pathology in exercise test, n (%)	6 (33.3)	7(2.0)	<0.001
Pathological T wave morphology, n (%)	3 (16.6)	3(0.8)	0.003
VES, n (%)	9 (50)	37(10.7)	<0.001
Ventricular tachycardia, n (%)	8(44.4)	2(0.5)	<0.001
Mean heart rate (beats/min)	78.9 (9.4)	80.6 (11.2)	0.31

ARVD, arrhythmogenic right ventricular dysplasia; ECG, electrocardiogram; VES, ventricular extrasystole. Note: Pathological T-wave morphology included T-wave inversion, biphasic or notched T waves, diffuse flat T waves, T-wave alternans, bifid T waves, and prolonged Tpeak–Tend interval.

## Data Availability

The data that support the findings of this study are not publicly available due to the fact that their containing information could compromise the privacy of research participants. However, data are available from the corresponding author upon reasonable request.

## References

[1] Martínez-Barrios E., Cesar S., Cruzalegui J., Hernandez C., Arbelo E., Fiol V., Brugada J., Brugada R., Campuzano O., Sarquella-Brugada G. (2022). Clinical Genetics of Inherited Arrhythmogenic Disease in the Pediatric Population. Biomedicines.

[2] González A., Aurlien D., Haugaa K.H., Taubøll E. (2018). Epilepsy in patients with long QT syndrome type 1: A Norwegian family. Epilepsy Behav. Case Rep..

[3] Moore B.M., Roston T.M., Laksman Z., Krahn A.D. (2025). Updates on inherited arrhythmia syndromes (Brugada syndrome, long QT syndrome, CPVT, ARVC). Prog. Cardiovasc. Dis..

[4] Nehme R.D., Sinno L., Shouman W., Ziade J.A., Ammar L.A., Amin G., Booz G.W., Zouein F.A. (2025). Cardiac Channelopathies: Clinical Diagnosis and Promising Therapeutics. J. Am. Heart Assoc..

[5] Martínez-Barrios E., Grassi S., Brión M., Toro R., Cesar S., Cruzalegui J., Coll M., Alcalde M., Brugada R., Greco A. (2023). Molecular autopsy: Twenty years of post-mortem diagnosis in sudden cardiac death. Front. Med..

[6] Remme C.A. (2023). SCN5A channelopathy: Arrhythmia, cardiomyopathy, epilepsy and beyond. Philos. Trans. R. Soc. B Biol. Sci..

[7] Heron S.E., Hernandez M., Edwards C., Edkins E., Jansen F.E., Scheffer I.E., Berkovic S.F., Mulley J.C. (2010). Neonatal seizures and long QT Syndrome: A cardiocerebral channelopathy?. Epilepsia.

[8] Zaccara G., Lattanzi S. (2019). Comorbidity between epilepsy and cardiac arrhythmias: Implication for treatment. Epilepsy Behav..

[9] Kligfield P., Gettes L.S., Bailey J.J., Childers R., Deal B.J., Hancock E.W., van Herpen G., Kors A., Macfarlane P., Mirvis D.M. (2007). Recommendations for the Standardization and Interpretation of the Electrocardiogram. Part I: The Electrocardiogram and Its Technology A Scientific Statement From the American Heart Association Electrocardiography and Arrhythmias Committee, Council on Clinical Cardiology; the American College of Cardiology Foundation; and the Heart Rhythm Society Endorsed by the International Society for Computerized Electrocardiology. J. Am. Coll. Cardiol..

[10] Steinberg J.S., Varma N., Cygankiewicz I., Aziz P., Balsam P., Baranchuk A., Cantillon D.J., Dilaveris P., Dubner S.J., El-Sherif N. (2017). 2017 ISHNE-HRS expert consensus statement on ambulatory ECG and external cardiac monitoring/telemetry. Ann. Noninvasive Electrocardiol..

[11] Fletcher G.F., Ades P.A., Kligfield P., Arena R., Balady G.J., Bittner V.A., Coke L.A., Fleg J.L., Forman D.E., Gerber T.C. (2013). Exercise Standards for Testing and Training. Circulation.

[12] Van Der Ree M.H., Vendrik J., Kors J.A., Wilde A.A.M., Tan H.L., Postema P.G. (2020). Provocation testing is irreplaceable to uncover or refute Brugada syndrome—An electrocardiographic validation study during ajmaline provocation. Eur. Heart J..

[13] Cohen M.I., Triedman J.K., Cannon B.C., Davis A.M., Drago F., Janousek J., Klein G.J., Law I.H., Morady F.J., Paul T. (2012). PACES/HRS Expert Consensus Statement on the Management of the Asymptomatic Young Patient with a Wolff-Parkinson-White (WPW, Ventricular Preexcitation) Electrocardiographic Pattern. Heart Rhythm..

[14] Yang Y., Lv T., Li S., Liu P., Gao Q., Zhang P. (2022). Utility of Provocative Testing in the Diagnosis and Genotyping of Congenital Long QT Syndrome: A Systematic Review and Meta-Analysis. J. Am. Heart Assoc..

[15] Gollob M.H., Redpath C.J., Roberts J.D. (2011). The Short QT Syndrome. J. Am. Coll. Cardiol..

[16] Scrocco C., Ben-Haim Y., Ensam B., Aldous R., Tome-Esteban M., Specterman M., Papadakis M., Sharma S., Behr E.R. (2024). The role for ambulatory electrocardiogram monitoring in the diagnosis and prognostication of Brugada syndrome: A sub-study of the Rare Arrhythmia Syndrome Evaluation (RASE) Brugada study. Europace.

[17] Vitali F., Brieda A., Balla C., Pavasini R., Tonet E., Serenelli M., Ferrari R., Delise P., Rapezzi C., Bertini M. (2021). Standard ECG in Brugada Syndrome as a Marker of Prognosis: From Risk Stratification to Pathophysiological Insights. J. Am. Heart Assoc..

[18] Aggarwal A., Stolear A., Alam M.M., Vardhan S., Dulgher M., Jang S.J., Zarich S.W. (2024). Catecholaminergic Polymorphic Ventricular Tachycardia: Clinical Characteristics, Diagnostic Evaluation and Therapeutic Strategies. J. Clin. Med..

[19] Marcus F.I., McKenna W.J., Sherrill D., Basso C., Bauce B., Bluemke D.A., Calkins H., Corrado D., Cox M.G., Daubert J.P. (2010). Diagnosis of arrhythmogenic right ventricular cardiomyopathy/dysplasia: Proposed Modification of the Task Force Criteria. Eur. Heart J..

[20] Sarquella-Brugada G., Martínez-Barrios E., Cesar S., Toro R., Cruzalegui J., Greco A., Díez-Escuté N., Cerralbo P., Chipa F., Arbelo E. (2024). A narrative review of inherited arrhythmogenic syndromes in young population: Role of genetic diagnosis in exercise recommendations. BMJ Open Sport. Exerc. Med..

[21] Badura K., Buławska D., Dąbek B., Witkowska A., Lisińska W., Radzioch E., Skwira S., Młynarska E., Rysz J., Franczyk B. (2024). Primary Electrical Heart Disease—Principles of Pathophysiology and Genetics. Int. J. Mol. Sci..

[22] Han H., Ha F.J., Teh A.W., Calafiore P., Jones E.F., Johns J., Koshy A.N., O’Donnell D., Hare D.L., Farouque O.M. (2018). Mitral Valve Prolapse and Sudden Cardiac Death: A Systematic Review. J. Am. Heart Assoc..

[23] Haugaa K.H., Haland T.F., Leren I.S., Saberniak J., Edvardsen T. (2016). Arrhythmogenic right ventricular cardiomyopathy, clinical manifestations, and diagnosis. Europace.

[24] Crotti L., Brugada P., Calkins H., Chevalier P., Conte G., Finocchiaro G., Postema P.G., Probst V., Schwartz P.J., Behr E.R. (2023). From gene-discovery to gene-tailored clinical management: 25 years of research in channelopathies and cardiomyopathies. Europace.

[25] Wilde A.A.M., Amin A.S., Postema P.G. (2022). Diagnosis, management and therapeutic strategies for congenital long QT syndrome. Heart.

[26] D’Ascenzi F., Anselmi F., Graziano F., Berti B., Franchini A., Bacci E., Ceccon C., Capitani M., Bonifazi M., Mondillo S. (2019). Normal and abnormal QT interval duration and its changes in preadolescents and adolescents practicing sport. EP Eur..

[27] Amirian A., Zafari Z., Dalili M., Saber S., Karimipoor M., Dabbagh Bagheri S., Fazelifar A.F., Zeinali S. (2018). Detection of a new KCNQ1 frameshift mutation associated with Jervell and Lange-Nielsen syndrome in 2 Iranian families. J. Arrhythm..

[28] Milman A., Gourraud J.B., Andorin A., Postema P.G., Sacher F., Mabo P., Conte G., Giustetto C., Sarquella-Brugada G., Hochstadt A. (2018). Gender differences in patients with Brugada syndrome and arrhythmic events: Data from a survey on arrhythmic events in 678 patients. Heart Rhythm..

[29] Skinner J.R., Winbo A., Abrams D., Vohra J., Wilde A.A. (2019). Channelopathies That Lead to Sudden Cardiac Death: Clinical and Genetic Aspects. Heart Lung Circ..

[30] Fitzgerald N., Lawley C., Morrish A., Tarca A., Marcondes L., Asakai H., Turner C., Skinner J. (2026). Catecholaminergic polymorphic ventricular tachycardia in children—Incidence and trends in detection, presentation and management. Arch. Dis. Child..

[31] Ji H.Y., Hu N., Liu R., Zhou H.R., Gao W.L., Quan X.Q. (2021). Worldwide prevalence of early repolarization pattern in general population and physically active individuals. Medicine.

[32] Watanabe H., Makiyama T., Koyama T., Kannankeril P.J., Seto S., Okamura K., Oda H., Itoh H., Okada M., Tanabe N. (2010). High prevalence of early repolarization in short QT syndrome. Heart Rhythm..

[33] Pappone C., Vicedomini G., Manguso F., Saviano M., Baldi M., Pappone A., Ciaccio C., Giannelli L., Ionescu B., Petretta A. (2014). Wolff-Parkinson-White Syndrome in the Era of Catheter Ablation. Circulation.

[34] Hanna E.B. (2014). Syncope: Etiology and diagnostic approach. Cleve Clin. J. Med..

